# Application of implementation mapping to develop strategies for integrating the National Diabetes Prevention Program into primary care clinics

**DOI:** 10.3389/fpubh.2023.933253

**Published:** 2023-04-26

**Authors:** William B. Perkison, Serena A. Rodriguez, Fernanda Velasco-Huerta, Patenne D. Mathews, Catherine Pulicken, Sidra S. Beg, Natalia I. Heredia, Pierre Fwelo, Grace E. White, Belinda M. Reininger, John W. McWhorter, Roshanda Chenier, Maria E. Fernandez

**Affiliations:** ^1^The University of Texas Health Science Center at Houston School of Public Health, Houston, TX, United States; ^2^Center for Health Promotion and Prevention Research, The University of Texas Health Science Center at Houston School of Public Health, Houston, TX, United States; ^3^Southwest Center for Occupational and Environmental Health, The University of Texas Health Science Center at Houston School of Public Health, Houston, TX, United States; ^4^The University of Texas Health Science Center School of Public Health, Dallas, TX, United States; ^5^The University of Texas Health Science Center School of Public Health, Brownsville, TX, United States; ^6^Institute for Implementation Science, The University of Texas Health Science Center at Houston, Houston, TX, United States

**Keywords:** underserved, implementation mapping, diabetes, prevention, primary care, prediabetes

## Abstract

**Background:**

Diabetes is considered one of the most prevalent and preventable chronic health conditions in the United States. Research has shown that evidence-based prevention measures and lifestyle changes can help lower the risk of developing diabetes. The National Diabetes Prevention Program (National DPP) is an evidence-based program recognized by the Centers for Disease Control and Prevention; it is designed to reduce diabetes risk through intensive group counseling in nutrition, physical activity, and behavioral management. Factors known to influence this program’s implementation, especially in primary care settings, have included limited awareness of the program, lack of standard clinical processes to facilitate referrals, and limited reimbursement incentives to support program delivery. A framework or approach that can address these and other barriers of practice is needed.

**Objective:**

We used Implementation Mapping, a systematic planning framework, to plan for the adoption, implementation, and maintenance of the National DPP in primary care clinics in the Greater Houston area. We followed the framework’s five iterative tasks to develop strategies that helped to increase awareness and adoption of the National DPP and facilitate program implementation.

**Methods:**

We conducted a needs assessment survey and interviews with participating clinics. We identified clinic personnel who were responsible for program use, including adopters, implementers, maintainers, and potential facilitators and barriers to program implementation. The performance objectives, or sub-behaviors necessary to achieve each clinic’s goals, were identified for each stage of implementation. We used classic behavioral science theory and dissemination and implementation models and frameworks to identify the determinants of program adoption, implementation, and maintenance. Evidence- and theory-based methods were selected and operationalized into tailored strategies that were executed in the four participating clinic sites. Implementation outcomes are being measured by several different approaches. Electronic Health Records (EHR) will measure referral rates to the National DPP. Surveys will be used to assess the level of the clinic providers and staff’s acceptability, appropriateness of use, feasibility, and usefulness of the National DPP, and aggregate biometric data will measure the level of the clinic’s disease management of prediabetes and diabetes.

**Results:**

Participating clinics included a Federally Qualified Health Center, a rural health center, and two private practices. Most personnel, including the leadership at the four clinic sites, were not aware of the National DPP. Steps for planning implementation strategies included the development of performance objectives (implementation actions) and identifying psychosocial and contextual implementation determinants. Implementation strategies included provider-to-provider education, electronic health record optimization, and the development of implementation protocols and materials (e.g., clinic project plan, policies).

**Conclusion:**

The National DPP has been shown to help prevent or delay the development of diabetes among at-risk patients. Yet, there remain many challenges to program implementation. The Implementation Mapping framework helped to systematically identify implementation barriers and facilitators and to design strategies to address them. To further advance diabetes prevention, future program, and research efforts should examine and promote other strategies such as increased reimbursement or use of incentives and a better billing infrastructure to assist in the scale and spread of the National DPP across the U.S.

## Introduction

Prediabetes is one of the most prevalent chronic health conditions diagnosed in the United States (U.S.), estimated to affect 88 million individuals (1). Nearly 40% of those diagnosed with prediabetes will likely be diagnosed with diabetes within 4 years (2). This progression can be largely prevented through behavioral lifestyle changes that incorporate a sustainable healthy diet and physical activity resulting in a 5–7% weight loss (2, 3). The National Diabetes Prevention Program (National DPP) is an effective, evidence-based lifestyle change program shown to reduce the incidence of diabetes (4, 5). The National DPP includes a 22-h curriculum delivered *via* group sessions over the course of 12 months and focuses on helping participants make healthy lifestyle changes including improving nutrition, physical activity, and psychological well-being to achieve sustainable weight loss (5, 6). Individuals eligible to participate in the National DPP are typically referred to the program by health care providers but they can also self-enroll (7).

Although the National DPP has shown to be effective in delaying diabetes diagnoses (8, 9), its widespread adoption and implementation have been hindered by multiple barriers (10–12). At the provider level, barriers include limited awareness of the program among clinic staff and/or healthcare providers, limited provider referrals to the program, and lack of provider buy-in (10–12). In their assessment of multi-level barriers to program implementation, Baucom et al. (12) identified clinicians’ lack of knowledge about the National DPP as the primary barrier to referring patients. At the clinic level, limited use of electronic health records (EHR) features to assist with referrals, lack of reimbursement or incentive structures to support National DPP referrals and delivery, and lack of health educators to deliver the program are impediments to wider adoption and implementation of the program (13). Patient-level barriers include time, cost, and inconvenient program locations (12). Raising provider and patient awareness about the National DPP and increasing “brand recognition” remains an important priority to increase participation in the program.

Investigators from The University of Texas Health Science Center at Houston School of Public Health Center for Health Promotion and Prevention Research and the Center for Quality Health IT Improvement at the School of Biomedical Informatics (hereafter referred to as UTHealth team) partnered with the Texas Department of State Health Services (DSHS) to carry out a five-year project funded by the Centers for Disease Control and Prevention (CDC). The goal was to use Implementation Mapping to design and implement strategies to implement diabetes prevention guidelines and the National DPP in primary care clinics located in the DSHS Public Health Region (PHR) 6/5S (Gulf Coast). This process has real-world applications that can guide healthcare institutions in their efforts to scale the National DPP in their communities.

## Methods

The UTHealth team first recruited primary care clinics to participate in the project and identified partner National DPP sites. The UTHealth team and clinic partners (hereafter “team”) then used Implementation Mapping, a systematic planning framework, to develop strategies to adopt, implement, and sustain a referral system to National DPP sites (14).

### Clinic recruitment

The UTHealth team recruited primary care clinics to participate in the project using purposeful sampling based on their location within the Texas DSHS PHR 6/5S and their previous relationship with the UTHealth Center for Quality Health IT Improvement. UTHealth team members (e.g., research coordinators, and quality improvement specialists) created a list of clinics in the selected public health region that were currently or had previously received quality improvement, data analysis, and reporting services from the Center for Quality Health IT Improvement. Clinics’ leadership staff from the identified clinics were contacted by phone and email and were provided with a brief overview of the project, including the goal of assisting clinics with National DPP implementation. Once a clinic indicated interest in participating, an introductory teleconference was scheduled with the clinic leadership team. During the introductory meeting, the DPP program was described, and clinic staff responded to unstructured questions to learn more about the clinic’s priorities and its overall diabetes prevention and management goals.

### Partnering with National DPP

The UTHealth team identified and recruited CDC-recognized National DPPs based on their coverage area within the Texas DSHS PHR 6/5S, ability to offer virtual classes, cost to participants, and ability to provide program materials in English and Spanish. As the initial step in the recruitment process, the UTHealth team created a list of CDC-recognized National DPPs registered on the CDC website located in the selected public health region. Additional National DPPs were identified in advertisements in the American Medical Association newsletter and through referrals from the funding agency. The UTHealth team reached out to each program to gauge their interest in partnering with one of the participating clinics. The recruitment process focused primarily on National DPP that could offer classes that could meet the needs of the clinics’ patient population who were primarily under or uninsured and Spanish-speaking. Thus, the selected National DPPS offered classes at no cost to the participants (i.e., their program was already funded by public or private grants) and had classes in English and Spanish. Furthermore, since this implementation started while social distancing restrictions were still in place due to the COVID-19 pandemic, we selected programs offering remote or in-person classes. The National DPPs selected who partner with the clinics were a City of Houston-sponsored program, a Silicon Valley-based program, and a local private practice.

### Strategy planning using implementation Mapping

Implementation Mapping incorporates theory, stakeholder input, and data to guide implementation strategy development (15). The process leads planners through five iterative tasks: (1) conduct a needs and assets assessment and identify program adopters, implementers, and maintainers; (2) identify adoption, implementation, and maintenance outcomes, performance objectives (i.e., specific tasks or sub-behaviors required to adopt, implement, and maintain a program), and determinants, and create matrices of change objectives (i.e., changes required in each determinant that will influence the achievement of each performance objective); (3) select evidence- and/or theory-based methods and identify or develop implementation strategies; (4) produce implementation protocol and materials; and (5) evaluate implementation outcomes (14).

#### Task 1: Conduct a needs and assets assessment and identify program adopters, implementers, maintainers, and champions

Leaders at the four participating clinics completed an online 56-item survey and 60-min interviews to assess: (1) awareness of National DPP; (2) barriers to National DPP adoption, implementation, and maintenance; (3) clinics’ approaches to prediabetes diagnosis and management; (4) the use of clinical decision support for chronic disease management and technological capabilities; (5) existing referral systems to external lifestyle change programs; and (6) use and capacity of the clinic’s EHR system. Clinic decision support (CDS) is any EHR tool designed to enhance decision-making in the clinical workflow. Tools may include alerts and reminders to care providers and patients, clinical guidelines, condition-specific order sets, focused patient data reports and summaries, documentation templates, diagnostic support, and contextually relevant reference information. Upon completion of the needs and assets assessment survey and interviews, the UTHealth team worked with each clinic to develop and sign a Memorandum of Understanding (MOU) indicating an intent to adopt the National DPP.

The team defined the following roles responsible for adopting and integrating National DPPs into clinic processes at each clinic site. A *program adopter* was defined as a clinic staff member with the decision-making authority to start using a National DPP program (i.e., clinic leadership) and/or a staff member (i.e., clinic administration) directly involved in deciding to set up program referral processes. A *program implementer* was a staff member (i.e., physician, nurse practitioner, physician assistant) responsible for making program referrals and/or a clinic administrator responsible for educating staff. A *program champion* (i.e., a health care provider or clinic administration) was an implementer that advocated for promoting the National DPP among other clinic staff (e.g., communicating with technical support personnel to ensure that EHR referral procedures were in place and fit the goal of being able to refer patients to a program in a timely manner). Finally, *program maintainers* (i.e., clinic leaders from administration, health care providers, and National DPP providers) were those who were responsible for ensuring that the program was maintained over time.

#### Task 2: Identify adoption, implementation, and maintenance outcomes, performance objectives and determinants, and create matrices of change objectives

In Task 2, the team stated the adoption, implementation, and maintenance outcomes, and performance objectives associated with each outcome. The overall goal is a statement that clinics intend to adopt, implement, and maintain a program while adoption, implementation, and maintenance outcomes are specific to each adopter, implementer, and maintainer. Performance objectives are the specific actions or sub-steps required to adopt, implement, and maintain the National DPP in each clinic (14). To create performance objectives, the team asked, “who needs to do what to ensure that the program is adopted?” with similar questions asked for implementation and maintenance.

Next, the UTHealth team identified determinants influencing adoption, implementation, and maintenance. Determinants answer the question why an adopter, implementer, or maintainer would complete performance objectives and outcomes (14). For example, “why would clinic leadership adopt the National DPP at their clinic?” The UTHealth team identified an initial list of determinants based on Task 1 data, a review of the literature, health behavior theories, and implementation and dissemination frameworks, and then provided clinic stakeholders with the list and solicited feedback to select final determinants. Stakeholders rated determinants based on perceived importance and changeability.

Finally, the team created a matrix of change objectives by crossing performance objectives (rows) with determinants (columns). Change objectives in each cell stated what needs to change in a determinant to achieve the performance objective and provided a blueprint for identifying, selecting, or developing implementation strategies (14).

#### Task 3: Select theory-based methods and identify implementation strategies

In Task 3, the team collaborated to identify evidence- and theory-based methods targeting determinants. Evidence- and theory-based methods are techniques influencing determinants and may work at the individual- and/or clinic-levels (14). Collaboration to identify methods included brainstorming, identifying previously successful methods in implementing organizational change at each clinic, and reviewing the literature. Next, the team operationalized methods as implementation strategies, the specific approaches to enhance National DPP adoption, implementation, and maintenance in participating clinics (14, 16, 17).

#### Task 4: Produce implementation protocols and materials

In Task 4, the team produced protocols and materials to facilitate National DPP adoption, implementation, and maintenance. Clinic action plans and supporting materials were developed and discussed during monthly TA calls to ensure the clinics’ feedback was incorporated. Clinic action plans delineated the implementation timeline. Supporting materials were developed and tailored to meet the needs of the clinics (e.g., staff, EHR capability, and patient population).

#### Task 5: Evaluate implementation outcomes

Data collection for evaluation is ongoing. Evaluation will include assessment of National DPP referrals *via* the EHR and adoption and implementation outcomes including program appropriateness, acceptability, feasibility, and fidelity measured *via* healthcare provider and clinic leadership surveys (15). Evaluation methods will include clinic leadership and healthcare provider surveys and document review of meeting notes, EHR screen captures, workflow/process flowcharts, and clinic policies.

## Results

### Clinic and National DPP partnerships

Four clinics meeting eligibility criteria agreed to participate. These included: Clinic A, a federally qualified health center (FQHC) with four clinic sites; Clinic B, a Rural Health Center (RHC); and Clinics C and D, two private community-based healthcare clinics. FQHCs are community-based health facilities eligible to receive federal funds because they provide affordable services to patients based on their ability to pay (18). RHCs are clinics that serve both private and publicly insured populations in rural, underserved areas; they can be for-profit or non-profit clinics (19). All participating clinics serve diverse patient populations and provide services to primarily under and uninsured patients with limited access to healthcare. The UTHealth team worked closely with stakeholders from each clinic including clinic leadership (e.g., chief executive officer, chief operations officer, chief medical officer, chief nursing officer); clinic administrators (e.g., technology/data analyst, practice administrator, practice manager); and health care providers (e.g., physicians, nurse practitioners, physician assistants).

The UTHealth team established partnerships with three National DPP, all of which were providing only virtual sessions as a result of the COVID-19 pandemic. The National DPPs were paired (i.e., the clinic needs matched with the program services) with clinics based on the capacity and preferences of the two partnering entities. For example, one clinic was paired with a local National DPP that offered face-to-face classes in English and Spanish reflecting the language needs of the clinic’s patient population.

### Implementation mapping

#### Task 1: Conduct a needs and assets assessment and identify program adopters, implementers, maintainers, and champions

##### Conduct a needs and assets assessment

Table 1 summarizes the results of the clinics’ needs assessment survey and interviews. Each clinic provided some form of patient education about diabetes prevention, although sources for materials differed by clinic. Screening for the risk of diabetes also varied by clinic, and only one clinic used clinical decision support to identify patients with prediabetes. Three of the four clinics were not aware of the National DPP or of its availability in their communities.

**Table 1:** Summary of the 2019 needs assessment survey and interview responses from clinics participating in the National Diabetes Prevention Program.

Key themes	Clinic A	Clinic B	Clinic C	Clinic D
Location	Rural	Urban	Urban	Urban
Clinic type	FQHC	FHQC	Private practice	Private practice
Patient population	7,500	21,254	6,000	1,000
Pre-diabetes education for patients	Education material provided includes materials from EHR, ADA, pharmaceutical companies, and counseling. No CHWs, but tech aides assist with patient management.	Education is provided by the MA and *via* pamphlets. Dieticians provide educational information and material on nutrition. Standard protocol with patients who have pre-diabetes is to provide education on lifestyle changes and referral to a dietician.	Education and instructions are given verbally by the physician.	Education handout was given *via* EHR.
Diabetes screening	Any patient at risk for diabetes is tested annually.	Any patient 40+ with risk factors of diabetes is tested.	Any patient at risk for diabetes is tested. No tools or algorithms are used for testing.	New patients are tested automatically at baseline.
Use of clinical decision support	Not applicable	Not applicable	Not applicable	Reminders for treatment
Awareness of the National DPP	No	Yes – did not make referrals	No	No
Awareness of local National DPP	No	Yes – did not make referrals	No	No
Provider-level barriers to referring patients to the National DPP	Perception that patients will not adhere to the National DPP. Perceived lack of time during appointments to discuss the National DPP and make referrals.	Perceived lack of time during appointments to use decision support tools, discuss the National DPP, and make referrals.	Lack of time during appointments. Perceived lack of time during appointments to discuss the National DPP and make referrals.	Perceived lack of time during appointments to use decision support tools, discuss the National DPP, and make referrals.
Perceived patient-level barriers to participating in the National DPP	Financial and time restraints. Patients low perceived risk.	Language transportation and childcare. Finding community resources.	No response	Finding community resources insurance consideration.

The data presented in this table was collected from the four participating clinics’ needs assessments completed in 2019.

ADA, American Diabetes Association; CHWs, community health workers; EHR, electronic health records; FHQC, federally qualified health center; MA, medical assistant; National DPP, National Diabetes Prevention Program; CDC, Centers for Disease Control and Prevention.

Clinic stakeholders identified the following two provider-level barriers to referring patients to the National DPP: (1) a perceived lack of time during appointments for the provider to use decision support tools, discuss the National DPP, and make referrals; and (2) the provider perception that patients will not adhere to the National DPP. The clinic stakeholders identified the following six perceived patient barriers to participating in a National DPP: (1) low understanding of diabetes risk perception; (2) language barriers; (3) financial and time constraints; (4) transportation difficulties; (5) childcare concerns; and (6) lack of health insurance.

Clinics reported using different EHRs including NextGen, Athena, Practice Fusion, and eClinicalWorks. Four clinics’ digital systems were not certified EHR products, had basic capabilities for setting appointments and billing, and were connected through the regional health information exchange and electronic provider-to-provider (P2P) referral networks. Most clinics used reminders for the treatment of diabetes as a CDS tool.

##### Identify program adopters, implementers, champions, and maintainers

Program adopters at clinics included clinic leadership (i.e., chief executive officer, chief operations officer, chief medical officer, and chief nursing officer). Program implementers included clinic administration staff (i.e., technology/data analyst, practice administrator, and practice manager), and healthcare providers (i.e., physicians making referrals, nurse practitioners, and physician assistants). Program champions were identified from both health care providers and clinic administration staff in each clinic. Finally, program maintainers were identified from leadership (i.e., chief executive officer, chief operations officer, chief medical officer, and chief nursing officer), clinic administration (i.e., technology/data analyst, practice administrator, and practice manager), and healthcare providers (i.e., physicians making referrals, nurse practitioners, and physician assistants).

#### Task 2: Identify National DPP adoption, implementation, and maintenance outcomes, performance objectives, and determinants and create matrices of change objectives

The identified outcomes were to adopt, implement, and maintain guidelines for diabetes prevention and the National DPP. Table 2 lists all adoption, implementation, and maintenance outcomes and performance objectives.

**Table 2:** Sample adoption, implementation, and maintenance outcomes and performance objectives.

Program: National DPP Setting: Clinic-based		
Target: role	Adoption, implementation, and maintenance outcomes	Performance objectives
Adopters		
Clinic leadership	Clinic leadership adopts National DPP to prevent diabetes among patients with prediabetes.	Partners with a CDC-recognized National DPP. Delineates the clinic’s National DPP referral goals. Approves legal agreement with National DPP. Designates a clinic program champion to spearhead the implementation of the National DPP referral process. Establishes reporting of participants who meet prediabetes criteria to National DPP.
Clinic administration	Clinic administration optimizes EHR to identify patients with prediabetes and refer them to the National DPP.	Optimizes EHR to facilitate the referral process. Joins the P2P network. Collaborates with EHR vendors to obtain the needed EHR updates and establish a patient identification process. Enables EHR identification of National DPP-eligible patients. Educates staff on EHR National DPP updates. Incorporates the National DPP referral process into the clinic’s workflow. Educates clinics staff about National DPP referral patient criteria. Establishes quality control to monitor the referral process.
Implementers		
Clinic administration	Clinic administration monitors the referral system.	Educate clinic staff about the National DPP workflow and make changes to improve productivity. Encourages health care providers to make patient referrals. Identifies gaps in data reporting. Conducts monthly reports of patients who meet prediabetes criteria for National DPP referral. Submits referrals data report to National DPP quarterly.
Health care provider	Health care provider makes referrals of patients with prediabetes to National DPP.	Reviews patient’s medical records. Identifies patients with prediabetes. Discusses National DPP referral with patients with prediabetes. Connects patients to the National DPP providers. Encourages patients to enroll in National DPP. Submits patient referral to National DPP in the EHR. Shares appropriate patient information with National DPP providers.
Program champion	Program champion promotes and educates other clinic staff about the implementation of National DPP.	Advocates for the implementation of National DPP. Motivates clinic health care providers to make National DPP referrals. Ensures that the EHR referral process is operational. Communicates with the National DPP provider to ensure referral feedback. Receives confirmation about patients’ National DPP referral status.
National DPP provider	National DPP provider delivers the National DPP to referred patients with prediabetes.	Coordinates how to receive patients’ referrals with the clinic. Pulls and reviews the database of eligible National DPP patients from the clinic EHR continuously. Coordinates logistics for hosting introductory sessions and National DPP classes throughout the year-long program. Motivates patients to promote adherence to the National DPP program. Provides enrollment and outcome feedback to the clinic.
Maintainers		
Clinic leadership	Clinic leadership maintains contractual /data agreements with National DPP providers.	Ensures that the contract is up to date and renews data agreement with National DPP as needed. Monitors fidelity of the referral system.
Clinic administration	Clinic administration consistently monitors the National DPP referral system.	Updates EHR as needed. Continues to review patient outcomes on a regular basis. Collects referral data and reports to providers. Providers continue guidance and training for current and new staff on completing referrals.
National DPP provider	National DPP provider maintains the delivery of the program to patients with prediabetes referred to from clinic.	Coordinates ongoing enrollment of new National DPP cohorts from patients’ referrals. Works with the clinic to continue providing patient status updates.

This table shows a sample of the adoption, implementation, and maintenance outcomes and performance objectives selected for the implementation of the National DPP.

CDC, Centers for Disease Control and Prevention; National DPP; National Diabetes Prevention Program; EHR, electronic health records.

Implementers: clinic administration, health care providers, program champions, and National DPP providers. Maintainers: identified included clinic leadership, clinic administration, and National DPP providers.

Healthcare providers: physicians making referrals, nurse practitioners, and physician assistants.

Program champion: health care providers or clinic administration.

Clinic leadership: chief executive officer, chief operations officer, chief medical officer, and chief nursing officer.

Clinic administration: technology/data analyst, practice administrator, and practice manager.

National DPP provider: lifestyle change coach and program administrator.

Adoption, implementation, and maintenance determinants that clinic stakeholders considered important and changeable included those from the Social Cognitive Theory (20) and Interactive Systems Framework (21). These included: stakeholder and providers’ attitudes toward the importance of diabetes prevention, knowledge about the program, perceived severity of failing to refer prediabetic patients, perceived program benefits, perceived program effectiveness, staff capacity and motivation to overcome barriers, and staff capacity and motivation to implement the program. The team crossed all determinants with performance objectives to create change objectives. Tables 3, 4 provide example matrices of change objectives for National DPP adoption and implementation in clinics.

**Table 3:** Sample matrices of change objectives for the adoption of the National Diabetes Prevention Program among the participating clinics in Texas, United States.

Adoption outcome: Clinic leadership adopts National DPP to prevent diabetes among patients with prediabetes.				
Performance objectives	Knowledge	Perceived severity	Attitudes	Perceived benefits
**PO1**. Clinic leadership partners with a CDC-recognized National DPP.	**K1a**. Describe the steps for partnering with a National DPP provider.	**PS1a**. Understand that adopting the National DPP will decrease patients’ risk of developing diabetes.	**A1a**. Believe that lifestyle change programs can help patients with prediabetes decrease the risk of developing diabetes.	**PB1a**. Expresses that referring patients with prediabetes to the National DPP will decrease their risk of developing diabetes.
**PO2**. Clinic leadership delineates the clinic’s National DPP referral goals.	**K2a**. List the number of patients with diabetes and prediabetes (at risk). **K2b**. Describes the expected change/patient outcomes in preventing diabetes.	**PS2a**. Understand the importance of setting goals for referrals to track referral outcomes. **PS2b**. Understand that setting achievable referral goals will help the clinic prevent diabetes.	**A2a**. Express a positive attitude about setting referral goals to promote referrals to the National DPP.	**PB2a**. Recognize that identifying clinic-wide referral goals will help providers make more informed decisions about making referrals. **PB2b**. Understand that by identifying referral goals, they will be able to track success.
**PO3**. Clinic leadership reviews and approves legal agreement (MOU) with National DPP.	**K3a**. Lists terms of the agreement. **AK3b**. Describes what the partnership will entail in detail.	**PS3a**. Perceives that the National DPP partnership will help the clinic prevent diabetes.	**A3a**. Believes that the MOU will establish guidelines and scope work of the relationship with the National DPP.	**PB3**. Expresses the need to have an MOU to guide the partnership successfully and provide accountability.
**PO4**. Clinic leadership designates a clinic program champion to spearhead the implementation of the National DPP referral process.	**K4**. Acknowledge that the program champion can successfully lead the clinic’s National DPP referral process.	**PS4a**. Believe that the program champion understands that the National DPP referral process fits the clinic’s diabetes management goals.	**A4a**. Express that the program champion will acknowledge the benefits of the adoption of National DPP.	**PB4**. Recognize that the program champion will support the National DPP referral efforts.
**PO5**. Clinic leadership establishes reporting of participants who meet prediabetes criteria to the National DPP.	**K5a**. List criteria for diagnoses of prediabetes. **K5b**. Understand how to pull patients with prediabetes based on lab values. **K5c**. Describe inclusion and exclusion criteria for National DPP participation.	**PS5a**. Understand the complications patients may experience if they progress from prediabetes to diabetes. **PS5b**. Understand that diabetes is a serious disease that can be prevented through early intervention in identified patients.	**A5a**. Express a positive attitude about pulling information of patients with prediabetes.	**PB5a**. Recognize that identifying patients with prediabetes will help the patients and providers make more informed decisions about the patient’s health. **PB5b**. Understand that by identifying patients with prediabetes, they will now be able to connect them with useful educational resources.

This table shows a sample of the performance objectives for the adoption of the National DPP program based on the determinants from the Social Cognitive Theory (SCT) and Health Behavior Model (HBM).

CDC, Centers for Disease Control and Prevention; EHR, electronic health records.

Healthcare providers: physicians making referrals, nurse practitioners, and physician assistants.

Program champion: health care providers or clinic administration.

Clinic leadership: chief executive officer, chief operations officer, chief medical officer, and chief nursing officer.

Clinic administration: technology/data analyst, practice administrator, and practice manager.

National DPP provider: lifestyle change coach and program administrator.

**Table 4:** Sample matrices of change objectives for the implementation of the National Diabetes Prevention Program among participating clinics in Texas, United States.

Implementation outcome: Health care provider makes referrals of patients with prediabetes to the National DPP.				
Performance objectives	Perception and awareness	Outcome expectations	Feedback and reinforcement	Interorganizational relationships
**PO1**. Health care providers reviews patients’ medical records.	**PA1a**. Perceive that reviewing patient records is necessary and important to identify and properly refer patients with prediabetes to National DPP.	**OE1a**. Expect that review of patient records is necessary and important to make a proper National DPP referral. **OE1b**. Expect that reviewing the patient’s health records will be of value for making the referral to National DPP.	**FR1a**. Express that reviewing patient records will result in increased referral of patients with prediabetes to the National DPP.	**IR1a**. Acknowledge the benefits of other clinic members reviewing the medical records pre-appointment.
**PO2**. Health care providers identify patients with prediabetes (at risk of diabetes).	**PA2a**. Perceive that identifying patients with prediabetes is an important step toward making referrals to the National DPP. **PA2b**. Perceives that understanding the inclusion and exclusion criteria of DPP participation is key to making a referral to the National DPP.	**OE2a**. Expects that the identification process will help refer patient population at risk of diabetes. **OE2b**. Expects that lab values are important to identify patients susceptible to diabetes.	**FR2a**. Expresses the importance of identifying patients at risk of diabetes. **FR2b**. Expresses that diabetes is a serious disease that can be prevented through early identification and prevention.	**IR2a**. Acknowledges the impact of identifying patients with prediabetes to help the clinic’s efforts to prevent diabetes. **IR2b**. Recognizes that screening patients for prediabetes will help them and the clinic staff to refer patients to the National DPP.
**PO3**. Health care providers discusses National DPP referral with the patient	**PA4a**. Perceive the success of the National DPP program in preventing diabetes. **PA4b**. Acknowledge the ability to discuss the National DPP referral with patients.	**OE4a**. Expect that the patient may not trust the National DPP program without a conversation with their provider.	**FR4a**. Express positive attitude about discussing the National DPP referral with the patient.	**IR4a**. Recognize that provider-patient communication increases trust in the patient for the National DPP. **IR4b**. Recognize that the discussion with the patient may increase their likelihood of attending and fully adhering to the National DPP.
**PO5**. Health care providers encourage patients to enroll in the National DPP.	**PA5a**. Feel that the National DPP referral process is necessary and important for the success of the intervention and patient enrollment.	**OE5a**. Expect that National DPP referral will incentivize patients to buy-in the enrollment process.	**FR5a**. Believe that encouraging patients to enroll in the National DPP will enhance patient enrollment.	**IR5a**. Recognize that encouraging patients to enroll in the National DPP may help patients enroll in the program.
**PO6**. Health care providers submit patient referrals to National DPP in the EHR.	**PA6a**. Perceive that submitting patient referrals is easy and important for patients to join the National DPP to prevent diabetes.	**OE6a**. Expect that submitting referral is key for patients to enroll in the National DPP. **OE6b**. Expect that submitting referrals will help patients connect with the National DPP.	**FR6a**. Express that submitting patient referrals will result in increased enrollment of patients with prediabetes in the National DPP.	**IR6a**. Acknowledge that submitting referrals will facilitate patient enrollment to prevent diabetes.
**P07**. Health care providers share appropriate patient information (contact information and lab work) with National DPP providers.	**PA7a**. States the importance of sharing patient information with the National DPP to support enrolment. **PA7b**. Acknowledge the importance of submitting the patient’s information as part of the referral process to the National DPP.	**OE7a**. Expect that sharing patient information will ensure timely program enrollment.	**FR7a**. Express satisfaction about sharing patients’ information with the National DPP as part of the referral process.	**IR7a**. Recognize that providing the patient’s information will help the National DPP communicate with patients. **IR7b**. Recognize that providing the patient’s information will ensure eligibility to the National DPP.

This table shows a sample of the performance objectives and determinants for the implementation of the National DPP program based on the Interactive Systems Framework (ISF) for Dissemination and Implementation.

CDC, Centers for Disease Control and Prevention; EHR, electronic health records.

Healthcare providers: doctors making referrals, nurse practitioners, and physician assistants.

Program champion: health care providers or clinic administration.

Clinic leadership: chief executive officer, chief operations officer, chief medical officer, and chief nursing officer.

Clinic administration: technology/data analyst, practice administrator, and practice manager.

National DPP provider: lifestyle change program and program administrator.

#### Task 3: Select theory-based methods and identify implementation strategies

The team identified three primary evidence- and theory-based methods to influence determinants: enhancing network linkages; participatory problem solving, providing technical assistance, facilitation, goal-setting, framing, tailoring, and guided practice.

Methods were operationalized as specific implementation strategies to increase National DPP adoption, implementation, and maintenance. These included: (1) developing and distributing providing education materials; (2) monthly meetings between the clinic staff, the National DPP provider, and the UTHealth team; (3) changing clinic records systems to include an EHR-based referral system between clinics and partner National DPPs; and (4) provider-to-provider mentoring. Table 5 depicts determinants, linked theoretical methods, and implementation strategies operationalizing the methods.

**Table 5:** Sample matrices of change objectives for the maintenance of the National Diabetes Prevention Program among participating clinics in Texas, United States.

Maintenance outcome: Clinic administration consistently monitors the National DPP referral system.				
Performance objectives	Perception and awareness	Outcome expectations	Feedback and reinforcement	Interorganizational relationships
**PO1**. Clinic administration updates EHR as needed.	**PA1a**. Acknowledge that the program champion can successfully lead the clinic’s National DPP referral process.	**OE1a**. Believe that the program champion understands that the National DPP referral process fits the clinic’s diabetes management goals.	**FR1a**. Express that the program champion will acknowledge the benefits of the adoption of National DPP.	**IR1a**. Recognize that the program champion will support the National DPP referral efforts.
**PO2**. Clinic administration continues to review patient outcomes on a regular basis.	**PA4b**. Describes referring patients with prediabetes to National DPP as a good fit for the clinic to decrease prediabetic patients’ risk of developing diabetes.	**OE3a**. Expects that incorporating the National DPP referral process into the clinic’s workflow will contribute to the successful implementation of the National DPP referral.	**FR3a**. Recognize that incorporation of the National DPP into the clinic’s workflow will result in increased referrals to National DPP.	**IR3**. Recognize that incorporating the National DPP workflow can help healthcare providers and other clinic staff complete the necessary steps to identify new and existing patients with prediabetes.
**PO3**. Clinic administration collects referral data and reports to providers.	**PA4a**. Perceive the success of the National DPP program in preventing diabetes. **PA4b**. Acknowledge the ability to discuss the National DPP referral with patients.	**OE4a**. Expect that the patient may not trust the National DPP program without a conversation with their provider.	**FR4a**. Express positive attitude about discussing the National DPP referral with the patient.	**IR4a**. Recognize that provider-patient communication increases trust in the patient for the National DPP. **IR4b**. Recognize that the discussion with the patient may increase their likelihood of attending and fully adhering to the National DPP.
**PO4**. Clinic administration provides continues guidance and training for current and new staff on completing referrals.	**PA4**. Describes resources and the importance for continuing provider about the DPP.	**OE4**. Expects that prioritizing continuing education will help current and new providers stay up to date with referral protocols for identifying and refer patients to the National DPP.	**FR4**. Expresses that continuing training is important to keep up with guidelines and help new staff gain the knowledge needed to make referrals.	**IR4**. Recognizes the importance of continuing education to maintain the referral numbers/process when new staff are hired.

#### Task 4: Produce implementation protocol and materials

Once the referral network was established between the clinics and the National DPP providers, the partnering program began to contact and enroll participants. Through participatory planning sessions with each clinic and its assigned program provider, we identified the need for introductory sessions, referred to as “Session 0,” to help participants become familiar with the virtual platform used by the National DPPs. The partnering program established a virtual meeting, assigned participants to 15-min time slots, and provided guidance to the team on what aspects of the program were critical to communicate to participants. The clinic’s program champion, the program’s lifestyle change coaches, and the UTHealth team facilitated Session 0 by introducing participants to the National DPP, connecting them to their coach, and answering any questions about the virtual platform (Table 6).

**Table 6:** Example determinants, theoretical methods, and implementation strategies.

Implementation outcome:Health care provider makes referrals of patients with prediabetes to the National DPP.		
Determinants	Methods (Theory)	Implementation strategies
Perception and awareness Outcome expectations	**Modeling** (Social cognitive theory; diffusion of innovations theory) **Framing** (Protection motivation theory) **Tailoring** (communication-persuasion matrix) **Discussion** (elaboration likelihood model) **Goal-setting** (Goal-setting theory) **Feedback** (Theories of learning; social cognitive theory). **Guided practice** (Social cognitive theory)	**Develop and distribute tailored materials** Educational materials include salient, gain-framed messages highlighted: National DPP eligibility criteria and policies. EHR referral pathways Models of clinics implementing National DPP highlighted: National DPP providers discussing the importance of submitting patient referrals. How other clinics prioritize National DPP referrals and integrate the process in their current workflows. Testimonials from health care provider about the impact of the National DPP. Thank you notes to providers including a message of support for their referral's effort and the number of referrals made each quarter. Training materials included: Walkthrough presentations and handouts illustrate proper identification of patients to promote diabetes prevention and referral submission Reminder materials included: Flyer with diabetes risk factors, eligibility criteria, and program details. The flyers also included the National DPPs contact information and a message about the National DPP benefits from a participant's point of view and a gain-framed message (“Refer patients at risk of diabetes to the National DPP to reduce their risk of developing type 2 diabetes.”). **Monthly meetings between the clinic staff (e.g., leadership, administration, and program champion), National DPP and the UTHealth team to share knowledge and relay clinical data to providers.** Presentations and discussions to: Describe how to conduct referrals, including the use of decision support tools and benefits on patient outcomes. Discuss clinics’ diabetes prevention efforts, number of referrals made. Review patient records and referral numbers to identify opportunities for improvement. **Provider-to-provider mentoring** Meetings to give feedback on the progress of the providers' goals and referrals.
Interorganizational relationships	**Discussion** (Elaboration likelihood model) **Participatory problem solving** (Organizational development theories; social capital theory; models of community organization). **Enhancing network linkages** (Social networks and social support theory)	**Monthly meetings included the National DPP, the clinic staff (e.g., leadership, administration, and program champion), and the UTHealth Team.** Regular interaction between the National DPP, the clinic staff (e.g., leadership, administration, and program champion), and the UTHealth Team facilitated: Rapport and linkage building between teams. Troubleshooting as adoption or implementation barriers occurred. **Change clinic records systems to include EHR-based referral system between clinics and partner National DPP.** Updates/changes made to the clinics and National DPP EHR included: Connecting the health center EHR and the National DPP into the same network. Establishing direct messaging between the clinic and the National DPP to facilitate the referral process. Integrating lab results into the clinics EHR. **Promote network weaving by partnering the clinic with local food bank.** Facilitate integration of food bank services with the National DPP and clinics.
Feedback and reinforcement	**Technical Assistance** (TA) (Organizational development theories; diffusion of innovations theory; social capital theory; models of community organization)	**Centralized monthly technical assistance meetings with the National DPP, the clinic staff (e.g., leadership, administration, and program champion), and the UTHealth team.** Monthly meetings included: Training on how to use EHR-based referral system, benefits of using CDS to facilitate referrals Support and troubleshooting for EHR-based referral system Assistance with EHR/CDS optimization and workflows Discussions about the importance of reviewing and interpreting data trends on a continuous basis.

This table shows a sample of the methods and practical applications for environmental outcomes for clinics.

CDS, clinical decision support; EHR, Electronic Health Records; National DPP, National Diabetes Prevention Program; TA, Technical Assistance.

During planning sessions with the clinics, the team identified a need for materials to educate and inform patients and healthcare providers about the National DPP and the importance of program referrals. Collaborating with each clinic, the team developed National DPP referral policies, workflows, flyers and posters. Clinical workflows delineated who did what during the rooming, identification, referral, and follow-up process of patients eligible to the National DPP. Clinical pathways were captured during one-on-one TA calls with the clinic’s EHR specialist and a step-by-step document of the EHR referral process was shared with the clinic staff to orient providers making referrals using the clinics EHR. The flyers and posters were displayed on the clinics’ websites and within the clinics’ waiting and exam rooms. Flyers for providers included messaging about National DPP eligibility criteria and the selected National DPP provider(s) that had partnered with the clinic. In contrast to provider flyers, patient flyers provided an overview of the program and prompted them to speak with their health care provider about the program. While creating these materials, the team focused on integrating messaging that would address the change objectives in the matrices. For instance, an infographic was developed for clinic staff to use and post on their intranet that prompted providers to ask, “Are your patients at risk for diabetes?” and then prompted them to act with the call to action, “Refer patients at risk of diabetes to the National DPP to reduce their risk of developing type 2 diabetes.” Which was reinforced with the eligibility criteria of the program and a description of the benefits provided by the program. All of these developed protocol documents and materials were co-created and clinic staff provided the final review and approval prior to implementation.

#### Task 5: Evaluate implementation outcomes

Evaluation is ongoing and future manuscripts will report National DPP referrals made, adoption outcomes, and implementation outcomes.

## Discussion

Successful integration of the National DPP into the U.S. healthcare system is critically needed to counter the rapidly rising incidence of diabetes nationwide. By utilizing the Implementation Mapping planning framework, our coalition of primary care clinics and National DPP providers implement strategies to implement diabetes prevention guidelines and the National DPP with the intent of improving the identification of people with prediabetes and refer them to CDC-recognized lifestyle change programs for Type 2 diabetes prevention.

Through systematic planning using Implementation Mapping, we designed implementation strategies to address barriers, build capacity, and create systems to foster the adoption and implementation (10–12). We chose evidence- and theory-based methods and practical applications to improve acceptance and uptake of the implementation.

In the present project, Implementation Mapping proved to be a useful, systematic approach for identifying POs centered around the multiple actor-specific tasks required to ensure proper integration of the National DPP into the four clinics’ workflows. The Implementation Mapping framework helped us map practical applications to address determinants needed to achieve the POs needed to promote and identify local National DPP providers, promote the program’s value to clinic patients and providers, and optimize EHR capabilities to effectively communicate referrals between clinics and National DPP providers.

### Strengths and limitations

An important strength of this project was the experience and background of a collaborative transdisciplinary team including engaged partners. Team members included those experienced in using Implementation Mapping to scale preventive health programs, and others skilled in providing technical assistance on EHRs and referral pathways for clinical use. This rich history of collaboration and capabilities were instrumental in building rapport and trust with the four participating clinics, and in facilitating culturally appropriate support and materials that were individualized for each of the clinics.

A limitation of the project was the design of the needs assessment. The original survey and interviews did not ask about the clinics’ level of readiness nor their capacity to adopt and implement the referral procedures that are necessary to refer patients to National DPP providers. The focus of the project was implementation and promotion of the National DPP referrals. However, gaps in knowledge of the readiness and capacity of the clinics likely impeded some of the actions that could be taken during the Implementation Mapping process (22). As a result, the UTHealth team suggested examining inner setting factors that impact the sustainability of the National DPP and future studies.

## Conclusion

Diabetes is among the most prevalent chronic diseases in the U.S. This condition has devastating impacts on the quality of life of patients, with these negative consequences ranging from premature death and coexisting morbidity from complications to loss of work productivity and high health care costs (15, 23, 24). Yet, identifying individuals who are at risk for diabetes (i.e., people with prediabetes and/or a history of gestational diabetes) and helping them lower this risk have not been priorities for many health systems, even though evidence-based programs like the National DPP are available to patients and are now reimbursable under Medicare and several state Medicaid plans (24). Emerging research on program implementation suggests that patient and health care providers limited knowledge of the National DPP, along with the difficulties in maintaining patient attendance, and the sustainability of referrals process to the National DPP have been barriers to the wider use of this program (12). The implementation strategies developed helped clinics overcome barriers by educating providers about the National DPP and its benefits on diabetes prevention, promoting patient education, and facilitating the use of EHRs (12).

Enrollment is just the first step in this process, and adherence is also critical. There is a need for studies that explore how to increase adherence and how implementation could include use of incentives. For example, the UTHealth team is currently piloting an intervention that includes participation incentives to better understand its effect on patient adherence to promote National DPP attendance (12). The program demonstrated how Implementation Mapping can be used to help clinics and National DPP providers overcome implementation barriers. In the long term, healthcare leaders can use experiences of programs such as these to expand and help improve the quality of National DPP delivery and to increase its access for patients who are at high risk of developing diabetes.

## Data availability statement

The original contributions presented in the study are included in the article/supplementary material, further inquiries can be directed to the corresponding authors.

## Ethics statement

The studies involving human participants were reviewed and approved by the Committee for the Protection of Human Subject University of Texas Health Science Center. The ethics committee waived the requirement of written informed consent for participation.

## Author contributions

WP directed research directly and helped study design and wrote manuscript. PM and FV-H project manager, wrote the manuscript, and reviewed extensively. CP project manager, wrote the manuscript, and reviewed. SB worked in project, wrote manuscript, and reviewed. NH designed the project, reviewed implementation mapping process, wrote the manuscript, and reviewed. SR reviewed implementation mapping manuscript. PF worked in project and reviewed. BR and JM reviewed manuscript. RC oversaw project and reviewed. MF and PI were responsible project and wrote and reviewed manuscript. GW a reviewer of the manuscript requested an extensive reorganization of the methods and discussion section of the manuscript. I played a key role in this organization process making the manuscript which included rewriting parts, adding additional literature citations and adding new dimensions to the discussion. All authors contributed to the article and approved the submitted version.

## Funding

This project was supported by the Centers for Disease Control and Prevention of the U.S. Department of Health and Human Services (HHS) as part of a financial assistance award totaling $3.8 million with 100 percent funded by CDC/HHS. The contents are those of the author(s) and do not necessarily represent the official views of, nor an endorsement, by Texas DSHS, CDC/HHS, or the U.S. Government. This project was also provided by the Southwest Center for Occupational and Environmental Health (SWCOEH), a NIOSH Education and Research Center, and awardee of (Grant No. 5T42) H008421 from the National Institute for Occupational Safety and Health (NIOSH)/ Centers for Disease Control and Prevention.

## Conflict of interest

The authors declare that the research was conducted in the absence of any commercial or financial relationships that could be construed as a potential conflict of interest.

## Publisher’s note

All claims expressed in this article are solely those of the authors and do not necessarily represent those of their affiliated organizations, or those of the publisher, the editors and the reviewers. Any product that may be evaluated in this article, or claim that may be made by its manufacturer, is not guaranteed or endorsed by the publisher.
