# Corrigendum: Application of implementation mapping to develop strategies for integrating the National Diabetes Prevention Program into primary care clinics

**DOI:** 10.3389/fpubh.2023.1330474

**Published:** 2023-11-21

**Authors:** William B. Perkison, Serena A. Rodriguez, Fernanda Velasco-Huerta, Patenne D. Mathews, Catherine Pulicken, Sidra S. Beg, Natalia I. Heredia, Pierre Fwelo, Grace E. White, Belinda M. Reininger, John W. McWhorter, Roshanda Chenier, Maria E. Fernandez

**Affiliations:** ^1^The University of Texas Health Science Center at Houston School of Public Health, Houston, TX, United States; ^2^Center for Health Promotion and Prevention Research, The University of Texas Health Science Center at Houston School of Public Health, Houston, TX, United States; ^3^Southwest Center for Occupational and Environmental Health, The University of Texas Health Science Center at Houston School of Public Health, Houston, TX, United States; ^4^The University of Texas Health Science Center School of Public Health, Dallas, TX, United States; ^5^The University of Texas Health Science Center School of Public Health, Brownsville, TX, United States; ^6^Institute for Implementation Science, The University of Texas Health Science Center at Houston, Houston, TX, United States

**Keywords:** underserved, implementation mapping, diabetes, prevention, primary care, prediabetes

In the published article, there was an error in the Funding statement. The funding statement for this project was:

“This project was supported by the Centers for Disease Control and Prevention of the U.S. Department of Health and Human Services (HHS) as part of a financial assistance award totaling $3.8 million with 100 percent funded by CDC/HHS. The contents are those of the author(s) and do not necessarily represent the official views of, nor an endorsement, by Texas DSHS, CDC/HHS, or the U.S. Government. This project was also provided by the Southwest Center for Occupational and Environmental Health (SWCOEH), a NIOSH Education and Research Center, and awardee of (Grant No. 5T42) H008421 from the National Institute for Occupational Safety and Health (NIOSH)/Centers for Disease Control and Prevention.”

The correct Funding statement appears below.

